# The Army Medical Bill

**Published:** 1907-12

**Authors:** 


**A Monthly Review of Medicine and Surgery.**

*EDITOR:*

WILLIAM WARREN POTTER, M. D.

All communications, whether of a literary or business nature, books for review and exchanges, should be addressed to the editor 238 DELAWARE AVE., BUFFALO, N. Y.

VOL. LXIII. DECEMBER, 1907. NO. 5

### The Army Medical Bill.

SHORTLY after the close of the Spanish war the JOURNAL began a campaign in the interests of the acting assistant surgeons of the medical department of the army with a view to securing official recognition for the men who gave their services and received merely relative rank as first lieutenants without actual standing as officers although they performed all the duties usually performed by a commissioned medical officer except as a member of courts martial. One of the suggestions made was the organisation of a medical reserve corps made up of acting assistant surgeons who served during the war and that they should be commissioned. The matter of forming a reserve corps of medical officers of experience has been provided for in the army medical reorganisation bill which is now before Congress and which has been persistently shoved back for two years now to make room for other bills, which in the opinion of the scientific politician who rules the House, were of more importance. There is a chance that the bill will be brought out of committee during the coming session and if it is, it will be acted upon favorably. It has received the approval of the Secretary of War and the President and yet, there is no certainty even now, that it will become a law this year unless the medical profession demands its passage. Just what the opposition consists of is hard to determine except it be the congenital disinclination on the part of the average political leader to do anything for medicine unless he happens to catch the eye of the gallery. This spirit is not confined to the distinguished speaker of the house by any means; although because he does possess it and has recently paraded it to the detriment of the medical department of the army, one of the most valuable and hardest worked branches of the government services, it shows up more conspicuously than it does in purely local struggles for medical aid. In Buffalo, at the present time the hospitals are begging for an advance of the rate paid by the city and county for ward patients; they are on their knee before the supervisors and the councilmen asking for an even break. They are not asking a rate sufficient to keep a city patient a week at a profit; they are merely begging, and with astonishing and pathetic humility, for just enough to properly feed and care for the city's patients without pecuniary loss. And while the hospitals are begging with pleading voice, with institutional deficit staring them in the face, the authorities are throwing money into public buildings with a careless sort of air and jockeying with bids for expensive furnishings for armories with hardly more than a tenative suggestion of economy.

This comparison is drawn simply to show that the average politician has medical views of a more or less distorted character. The main difficulty is that the medical profession never occupies the gallery to which the politician looks for approval.

The army medical department bill as stated has been before Congress two years and it has been held up by the speaker of the House and by him alone. It is a most desirable bill and it ought to be passed. The medical department of the army is woefully lacking in numerical strength. Its entire available force is so occupied with the purely necessary work of the department that it is not possible for the surgeon general to furnish medical officers for the examination of recruits at recruiting stations. The result is that prospective soldiers are enlisted by retired line officers after a cursory examination, sent to rendevous and there examined by a medical officer. It is safe to say that a large percentage of these recruits are rejected after a proper physical examination and the government loses just so much money paid out for the subsistence and transportation of the rejected soldier. That is one sample of the economy practised by the average politician. Were the bill now before Congress passed, the medical corps would be increased to meet the actual requirements of the service and in addition there would be established a medical reserve corps made up largely of men who have had experience as acting assistant surgeons; they would be commissioned first lieutenants and paid only when on actual duty. That would then do away forever with the anomalous, if not actually degrading, position now occupied by the contract surgeon which is even worse than that experienced by the acting assistant surgeon of the Spanish war. The latter at least was allowed to wear a uniform of the same kind worn by the commissioned officer, except that the collar ornaments and shoulder straps were of silver instead of gold.

When the title of acting assistant surgeon was abolished and the non-commissioned surgeon became officially a "contract surgeon" he was given permission, also officially, to wear a uniform but it was specified that he must wear on his collar the letters "C. S.," instead of the letters "U. S. A.," and the medical department cross permitted his more fortunate predecessor, the acting assistant surgeon who, while he did the work of a surgeon, was officially, so far as the average army officer was concerned, a sort of necessary hanger-on, because he was under contract; he did not have a commission. The commissioned medical officers of the army recognised his unenviable position and were his friends without exception. The facility with which the majority of acting assistant surgeons mastered the intricacies of the technical "paper work" of the army, the making of reports and official returns, is the best evidence that he had the friendship and assistance of the members of the army medical corps.

Surgeon General O'Reilly is a progressive man and the medical department has made great strides under his administration. He wants to see the bill passed, because it will make it possible for him to build up a medical corps which for efficiency and preparedness will be second to none in the world.

The medical profession can materially assist in forcing the bill forward. The JOURNAL can say positively that if it comes to a vote it will be passed and a great service done the country by giving the Surgeon General of the Army an efficient medical corps of sufficient numerical strength.

Every medical man should write to his congressman a personal letter requesting him to make a personal effort to bring the army medical bill before the House for action. Then the average politician who controls the destinies of bills may hear the voice of the gallery.

---

ELSEWHERE is published a synopsis of the meeting of the Military Surgeons of the United States at Jamestown, Va. The meeting was one of the most interesting ever held and action was taken on several matters of more than ordinary importance, one of which was the passage of a resolution looking to the commissioning of the acting assistant surgeons who served during the Spanish-American war and their discharge from the service the day following the date of the commission. This will establish the standing of a class of medical officers who did splendid service during the war and who occupied the anomalous position of being officers without rank or standing. The appointment of Dr. J. Carlisle DeVries as assistant secretary to aid Major James Evelyn Pilcher, the efficient and hard-working secretary of the

Association, is a recognition of the excellent work Dr. DeVries has done is the past few years in aiding Dr. Pilcher to get together material for the programs. The Military Surgeons Association is a strong body and its influence will be felt in congress when legislation affecting the medical services of the United States is pending.

---

A STATUE of the late Dr. Joseph Leidy, of Philadelphia, recognised as one of the most distinguished American scientists of the last century, was unveiled October 30, 1907, on the west plaza of City Hall. As president of the Academy of Natural Science, professor of human and comparative anatomy and zoology in the University of Pennsylvania and president of the Wagner Free Institute of Science, Dr. Leidy added immeasurably to the position these institutions hold in the world of science. He was born in Philadelphia in 1823 and died in 1891.

---

A CERTIFICATE of incorporation was filed November 1, 1907, with the Secretary of State at Albany, for the Russell Sage Institute of Pathology of New York City. Its objects are stated to be the encouragement of scientific research in lines of medicine and pathology, the investigation of the diseases and infirmities of advanced life and the maintenance of laboratories for these purposes. The directors are Edward G. Janeway, Theodore C. Janeway, D. Bryson Delavan, Simon Flexner and Graham Lusk, all of New York. The Institute will act as pathologist to the City Hospital and City Home on Blackwell's Island. Mrs. Sage made a special gift of $300,000 for the endowment of the institute last June, but it was not incorporated until the date above mentioned owing to the absence of several of the directors in Europe.

